# Enhancing Organ Allocation Efficiency: A Pilot Study Evaluating Artificial Intelligence-Assisted Assessment of Donor Kidney Pathology

**DOI:** 10.7759/cureus.83656

**Published:** 2025-05-07

**Authors:** Jeffrey Campsen, Yelina Kim, Tiffany Chen

**Affiliations:** 1 Surgery/Transplant, Donor Connect - Organ Procurement Organization, Murray, USA; 2 Artificial Intelligence, Techcyte, Orem, USA; 3 Pathology, Techcyte, Orem, USA

**Keywords:** artificial intelligence, kidney transplantation, machine learning, organ allocation, pathology

## Abstract

Purpose: The purpose of this study is to evaluate the effectiveness of an artificial intelligence (AI)-assisted review (AAR) system in improving diagnostic accuracy, efficiency, and concordance with expert assessments during the evaluation of donor kidney viability.

Methods: Sixty H&E-stained frozen-section kidney biopsy slides from explant kidneys obtained for organ donation were evaluated. A board-certified renal pathologist established ground truth (GT) through manual digital evaluation on the Techcyte Fusion Platform. The slides were independently reviewed by an AI algorithm, a board-certified pathologist (Reviewer 2 (R2)), and a board-certified transplant surgeon (Reviewer 1 (R1)). After a washout period, AI-assisted reads were performed. The performance of AAR and manual digital review (MDR) was compared to the GT for total and sclerotic glomeruli (SG) counts, as well as concordance with kidney viability thresholds (using a 20% SG cutoff rate). Secondary outcomes included comparisons of review times and concordance rates for AAR, MDR, and AI analysis alone with the GT.

Results: AAR demonstrated concordance with GT across parameters. For R1, coefficient of determination (COD) values for SG counts improved with AAR (0.833) compared to MDR (0.81). Agreement at the 20% SG threshold for kidney viability was 98.33% for both AAR and MDR. AAR reduced mean review times (minutes) by 54.83% compared to MDR, with per-slide review times decreasing from 17:09 (MDR) to 8:35 (AAR). Pearson correlation coefficients (PCC) and concordance correlation coefficients (CCC) for AAR were generally higher than MDR, particularly for the percentage of SG, indicating improved alignment with GT. Analyses revealed no systematic bias, with AAR aligning more closely with GT compared to MDR for both reviewers.

Conclusion: The Techcyte algorithm reduces review time while maintaining accuracy and concordance with experts, promoting AI adoption to improve workflow efficiency and expedite transplantation decisions.

## Introduction

Kidney transplantation remains the optimal treatment for end-stage renal disease, offering improved quality of life and survival compared to dialysis [1]. However, the critical shortage of donor organs remains a major challenge in the field of transplantation. In the United States alone, over 100,000 patients are currently awaiting a kidney transplant [2]. Cadaveric donor organs play a crucial role in addressing this shortage, but their utilization is often constrained by the need for rapid, accurate, and standardized assessment of organ quality.

The evaluation of cadaveric kidney donors traditionally relies on clinical data, laboratory values, and imaging studies. However, these methods may not provide a comprehensive and real-time evaluation of organ quality after donation, particularly concerning histopathological changes in the kidney tissue. Procurement biopsies offer critical intraoperative insights, but their interpretation presents several challenges. The urgency of organ donation, constrained by a typical cold ischemia time limit of 24 hours, necessitates expedited yet accurate pathological assessment. Additionally, the availability of experienced pathologists with expertise in donor kidney evaluation is often limited, particularly given the variability in procurement timing and location.

To overcome these limitations, we have developed an artificial intelligence (AI)-driven approach to enhance the interpretation of kidney biopsy slides from cadaveric organ donors. This study represents a collaboration between Techcyte, a company specializing in AI-powered digital pathology solutions, and DonorConnect, an organ procurement organization. Our goal is to integrate AI-assisted workflows that support pathologists in conducting rapid, standardized, and high-accuracy assessments of donor kidney biopsies, ultimately improving the efficiency and reliability of organ quality evaluation. 

The potential benefits of AI-assisted pathology in organ donation are multifaceted. By enabling rapid, consistent, and high-quality interpretations of biopsy slides, this approach may help to minimize cold ischemia time, enhance decision-making processes for organ allocation, and potentially increase the utilization of viable organs that might otherwise be discarded due to diagnostic uncertainty. Furthermore, the AI model can serve as an educational tool, assisting less experienced pathologists or transplant surgeons in recognizing critical histopathologic features in donor kidney biopsies.

This study aims to validate the effectiveness of this AI-assisted approach by comparing the performance of human readers (pathologists and transplant physicians) with and without AI assistance in interpreting cadaveric kidney biopsy slides. We hypothesize that AI-assisted evaluation will enhance diagnostic accuracy, improve interobserver consistency, and expedite biopsy interpretation, ultimately facilitating more informed decision-making in the organ donation and transplantation process.

By integrating cutting-edge AI technology with clinical and pathology expertise, this research has the potential to redefine organ assessment, addressing key limitations in donor kidney evaluation and expanding the pool of viable transplantable organs. As the demand for kidney transplants continues to rise, leveraging AI in donor evaluation represents a pivotal advancement toward optimizing transplant outcomes and saving more lives [3].

## Materials and methods

Study objective

This study validated the intended use of the Fusion Organ Donation - Kidney Algorithm (FOD-KA). The FOD-KA is an instance segmentation model based on the Mask R-CNN architecture. The model was trained on 4,000 labeled glomeruli across 96 slides. The model was trained using a learning rate of 0.02, a batch size of eight, and 10 epochs. Light cropping and flipping augmentations were applied. 

The primary objective was to assess whether the AI-assisted review (AAR) results were statistically equivalent to those of the unassisted manual digital review (MDR) when compared to the ground truth (GT) in identifying the number of globally sclerotic and non-globally sclerotic glomeruli (SG) in frozen-section biopsies of donor kidneys. 

Two reviewers were chosen; Reviewer 1 (R1) was a board-certified kidney transplant surgeon and Reviewer 2 (R2) was a general board-certified pathologist. These represented the type of potential clinicians that would be determining the quality of biopsies in the real world.

The secondary objectives of the study included comparing the time required to complete the MDR vs. the AAR, evaluating the concordance rates of both MDR and AAR with the GT, and determining the concordance rate of AI analysis alone, without manual review, with the GT. This study was approved by the University of Utah Institutional Review Board (IRB #IRB_00148714).

Intended use

The FOD-KA automates the annotation of areas of interest on digital images of kidney biopsy samples. It is intended as an assistive tool for pathologists in assessing the viability of kidneys from deceased donors for potential transplantation. The algorithm is intended for use with frozen-section kidney biopsy samples stained with hematoxylin and eosin (H&E).

The software identifies and classifies normal and globally SG within a whole slide image uploaded to the Fusion platform. The reviewer is presented with a viewer displaying pre-classified areas of interest and then verifies the numbers of normal and globally SG.

Study site

The Techcyte internal laboratory (Orem, Utah) served as the primary study site. The Techcyte laboratory was responsible for relabeling, scanning the glass slides, and uploading the digitized slides to the Techcyte cloud platform. All digital slide reviews were performed remotely across the United States. 

Sample collection

A total of 254 H&E-stained frozen-section kidney biopsy slides were retrospectively collected between January 2022 and January 2024 from the archives of an organ procurement organization. The slides were obtained from post-procurement kidney biopsies of deceased donors intended for transplantation, collected from approximately 20 hospitals across Utah, Wyoming, Idaho, and Colorado.

Sample condition

Due to the retrospective nature of the sample collection, specimen preparation was not standardized. Any frozen-section kidney biopsy slides prepared using the standard H&E-staining protocol were included, resulting in variability in slide quality. The original diagnoses and patient-identifying information were not available at the time of collection. 

Sample selection

The samples were reviewed by the study coordinator according to predetermined inclusion and exclusion criteria. A total of 60 samples were then randomly selected, following the College of American Pathologists’ Guidelines for validating whole slide imaging systems for diagnostic purposes [4].

Sample randomization protocol

Due to the unavailability of the final diagnostic information for the samples, a blinding protocol was not implemented. However, a randomization protocol was implemented to ensure that the assessment results between reviewers (R1 and R2) and review methods remained unknown to the reviewers (total 2 reviewers, GT - expert reviewer, AI model). 

**Figure 1:**
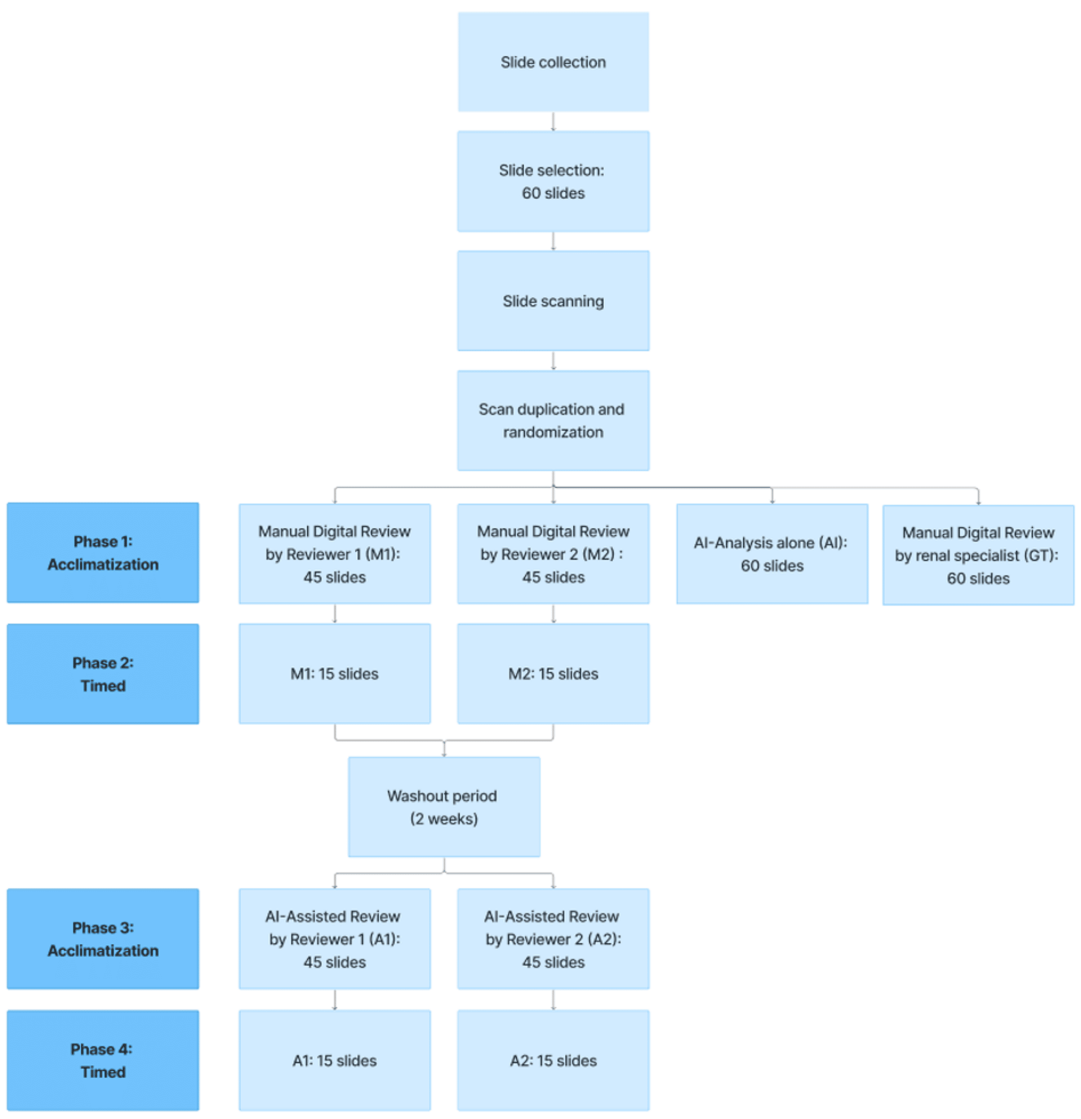
Study design

The study design, as shown in Figure 1, was conducted in four phases: Phase 1 was a non-timed acclimatization phase involving MDR of 45 samples; Phase 2 was a timed phase with MDR of 15 samples; Phase 3 was a non-timed acclimatization phase involving AAR of 45 samples; and Phase 4 was a timed phase with AAR of 15 samples.

A minimum two-week washout period was implemented between Phase 2 and Phase 3 to prevent reviewers from recalling previous assessments and influencing the subsequent evaluations. 

Manual digital review

The MDR referred to the process in which biopsy slides were scanned using the Grundium Ocus40 scanner and presented on the Techcyte Viewer. Reviewers manually identified globally sclerotic, defined as all glomeruli exhibiting global (complete) collapse of glomerular capillary walls and consolidation of the glomerular tuft by extracellular matrix, causing capillary luminal obliteration. Non-globally SG were also manually identified. Once the reviewers annotated glomeruli using the annotation tools available in the Techcyte platform, an automated enumeration tool calculated and displayed the final glomerular counts. 

Artificial intelligence-assisted review

The AAR was conducted using the same slides scanned with the Grundium Ocus40 scanner but analyzed by the FOD-KA for view. The FOD-KA pre-annotated, classified, and enumerated globally sclerotic and non-globally SG, presenting the results to the reviewers. Reviewers were instructed to verify the AI-generated classification, delete incorrectly classified glomeruli, reclassify mislabeled glomeruli, and identify and annotate glomeruli missed by the algorithm. 

Ground truth: A board-certified renal pathologist performed an MDR of all 60 slides. The results of this independent review served as the GT for statistical comparison. 

The statistical analyses performed included the coefficient of determination (COD), paired t-test, Pearson correlation coefficient (PCC), concordance correlation coefficient (CCC), and Bland-Altman analysis.

## Results

Primary analysis

To assess whether the results of the AAR were statistically equivalent to those of the MDR when compared to the GT, several parameters from both the AAR and MDR were analyzed for each reviewer. These parameters included the total glomeruli count, the globally SG count, and the percentage of globally SG.

For each parameter, the COD, calculated by squaring the PCC, was used to evaluate the strength of the linear relationship between each review method and the GT, as well as the proportion of variance in the GT explained by the AAR and MDR (Table 1). Higher COD values indicate that the method explains a greater proportion of the variability in the GT.

**Table 1:** For each reviewer, the COD was calculated to evaluate the strength of correlation with the GT MDR-GT vs. AAR-GT COD values. MDR, manual digital review; AAR, AI-assisted review; GT, ground truth; SG, sclerotic glomeruli; COD, coefficient of determination; R1, Reviewer 1; R2, Reviewer 2

	R1 - GT		R2 - GT	
	MDR-GT	AAR-GT	MDR-GT	AAR-GT
Total glomeruli count	0.918	0.904	0.862	0.853
SG count	0.810	0.833	0.643	0.569
% SG	0.876	0.903	0.749	0.752

To further evaluate whether the average deviation (bias) from the GT differed between AAR and MDR, paired t-tests were performed for each reviewer and parameter (Table 2). 

**Table 2:** For each reviewer, the paired t-test analysis was performed to compare AAR and MDR MD, mean difference; SD, standard deviation of mean difference; DF, degree of freedom; AAR, AI-assisted review; MDR, manual digital review; SG, sclerotic glomeruli; R1, Reviewer 1; R2, Reviewer 2

	R1: AAR vs. MDR					R2: AAR vs. MDR				
	MD	SD	t-statistic	DF	p-value	MD	SD	t-statistic	DF	p-value
Total glomeruli #	-3.5667	18.9472	-1.4581	59	0.1501	3.7333	12.5831	2.2982	59	0.0251
SG #	-0.3167	3.9251	-0.6249	59	0.5344	2.7500	5.8386	3.6484	59	0.0006
% SG	0.3106	3.6550	0.6582	59	0.5129	2.3125	5.4713	3.2739	59	0.0018

For R1, the AAR generally demonstrated higher COD values than the MDR, suggesting a stronger correlation with the GT. However, paired t-tests revealed no statistically significant difference in the mean measurements between AAR and MDR for any parameter. 

For R2, the MDR had slightly higher COD values than AAR for total glomeruli and SG counts, while the CODs for the percentage of SG were nearly identical (AAR=0.752, MDR=0.749). In contrast to R1, paired t-tests for R2 showed statistically significant differences in the mean measurements between AAR and MDR for all parameters. 

These findings suggest that reviewer-specific factors influence the agreement between AAR and MDR. While AAR may exhibit bias for individual reviewers, the inconsistent direction and magnitude of this bias across reviewers argue against a systemic bias introduced by AI assistance itself. Notably, even when AI assistance was employed, the rate of SG, a key metric in determining kidney transplant viability, remained stable across both methods, further supporting the conclusion that AI-assisted workflows do not introduce systemic bias.

Agreement with the ground truth for the 20% sclerotic glomeruli cutoff

To evaluate how each method aligned with the GT in terms of the 20% SG cutoff rate for viability assessment, each sample was assessed for agreement with the GT, and overall percentage agreement with the GT was calculated (Table 3).

**Table 3:** Agreement with GT: 20% glomeruli threshold MDR, manual digital review; AAR, AI-assisted review; GT, ground truth; R1, Reviewer 1; R2, Reviewer 2

AI alone	R1		R2	
	MDR	AAR	MDR	AAR
91.67%	98.33%	98.33%	93.33%	93.33%

The results demonstrated high consistency between MDR and AAR for both R1 and R2, indicating that AI assistance does not compromise diagnostic accuracy in assessing the 20% threshold for transplant viability.

Time comparison

To compare the efficiency of MDR vs. AAR, the review time per slide and per glomerulus was measured, and the percentage difference was calculated (Table 4).

**Table 4:** To compare the efficiency of MDR vs. AAR, the review time per slide and per glomerulus was measured, and the percentage difference was calculated MDR vs. AAR: review time comparison (minutes:seconds). MDR, manual digital review; AAR, AI-assisted review; R1, Reviewer 1; R2, Reviewer 2

	Per slide			Per glomerulus		
	MDR	AAR	% reduction	MDR	AAR	% reduction
R1	30:02	15:31	48.34%	00:15	00:08	46.67%
R2	04:16	01:39	61.33%	00:04	00:02	50.00%
Mean	17:09	08:35	54.83%	00:10	00:05	48.33%

Across both reviewers, AAR significantly reduced review time compared to MDR, demonstrating an increase in efficiency of approximately 55% per slide and 48% per glomerulus.

Concordance analysis

To compare the concordance rates of MDR, AAR, and AI analysis alone with the GT, two metrics were calculated: the PCC, which measures the linear correlation with the GT, and the CCC, which assesses overall agreement with the GT (Table 5; p<0.001).

**Table 5:** Across reviewers, PCC and CCC values compared the success rate of SG identification with the AAR, relative to the MDR PCC, Pearson correlation coefficient; CCC, concordance correlation coefficient; AAR, AI-assisted review; MDR, manual digital review, SG, sclerotic glomeruli

		AI alone	R1		R2	
			MDR	AAR	MDR	AAR
PCC	Total glomeruli #	0.947	0.958	0.951	0.929	0.923
	SG #	0.723	0.900	0.913	0.802	0.754
	% SG	0.835	0.936	0.950	0.865	0.867
CCC	Total glomeruli #	0.943	0.930	0.932	0.926	0.923
	SG #	0.723	0.854	0.876	0.737	0.567
	% SG	0.827	0.925	0.938	0.821	0.855

**Figure 2:**
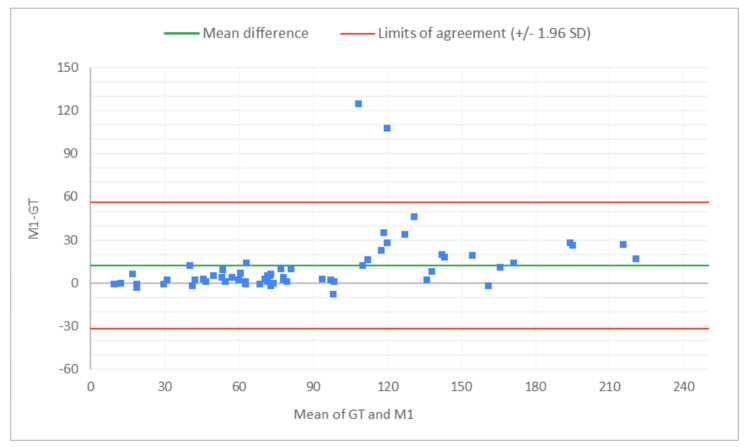
Bland-Altman plot for total glomeruli count: M1 (MDR of R1) vs. GT The x-axis represents the average of the two measurements, while the y-axis represents the difference between the two measurements. GT, ground truth; MDR, manual digital review; R1, Reviewer 1

**Figure 3:**
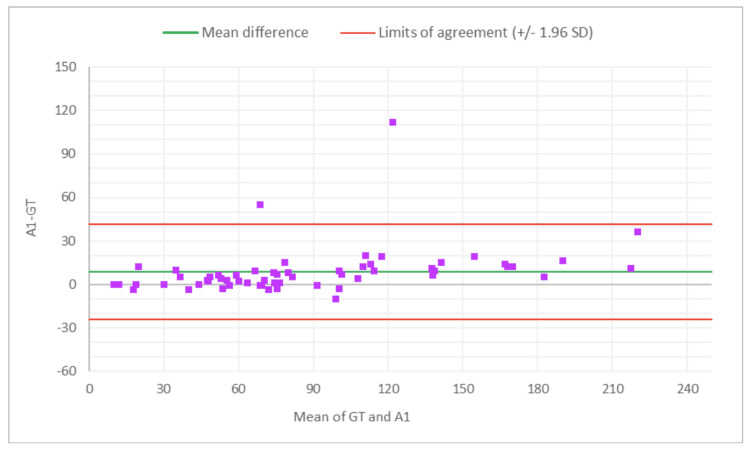
Bland-Altman plot for total glomeruli count: A1 (AAR of R1) vs. GT The x-axis represents the average of the two measurements, while the y-axis represents the difference between the two measurements. MDR, manual digital review; AAR, AI-assisted review; R1, Reviewer 1

**Figure 4:**
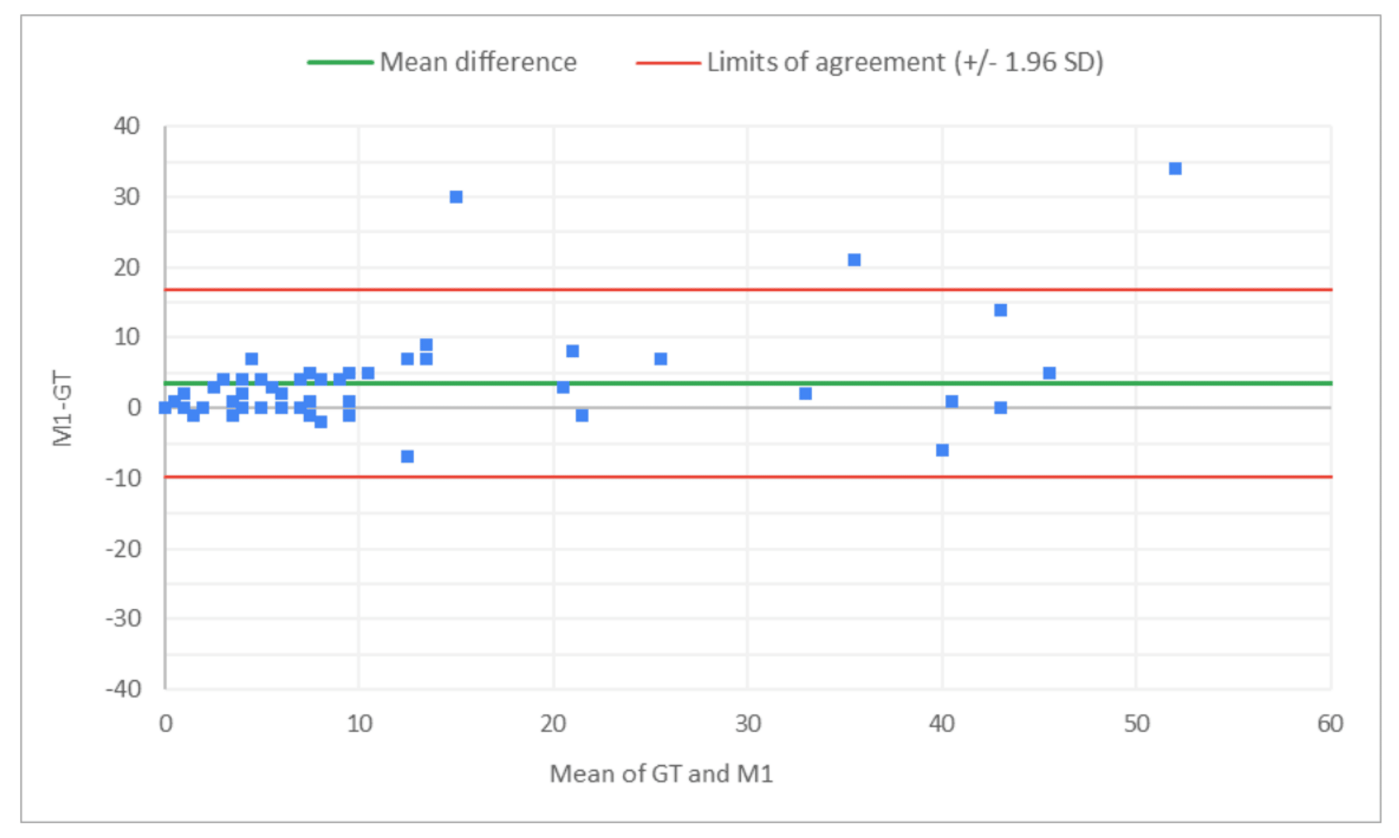
Bland-Altman plot for SG count: M1 (MDR of R1) vs. GT The x-axis represents the average of the two measurements, while the y-axis represents the difference between the two measurements. GT, ground truth; MDR, manual digital review; R1, Reviewer 1; SG, sclerotic glomeruli

**Figure 5:**
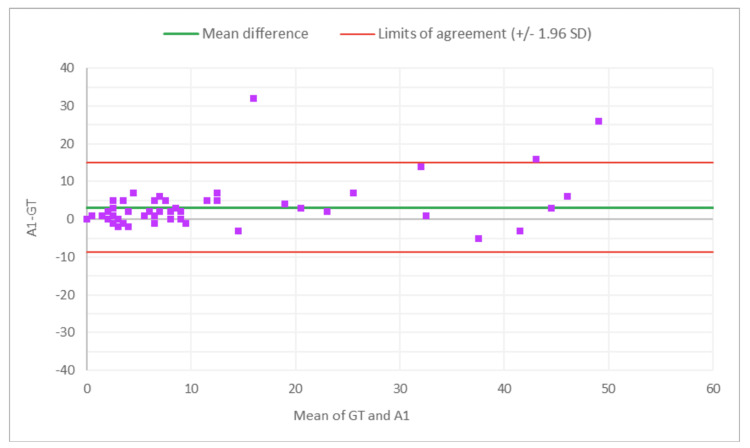
Bland-Altman plot for SG count: A1 (AAR of R1) vs. GT The x-axis represents the average of the two measurements, while the y-axis represents the difference between the two measurements. GT, ground truth; SG, sclerotic glomeruli; AAR, AI-assisted review; R1, Reviewer 1

**Figure 6:**
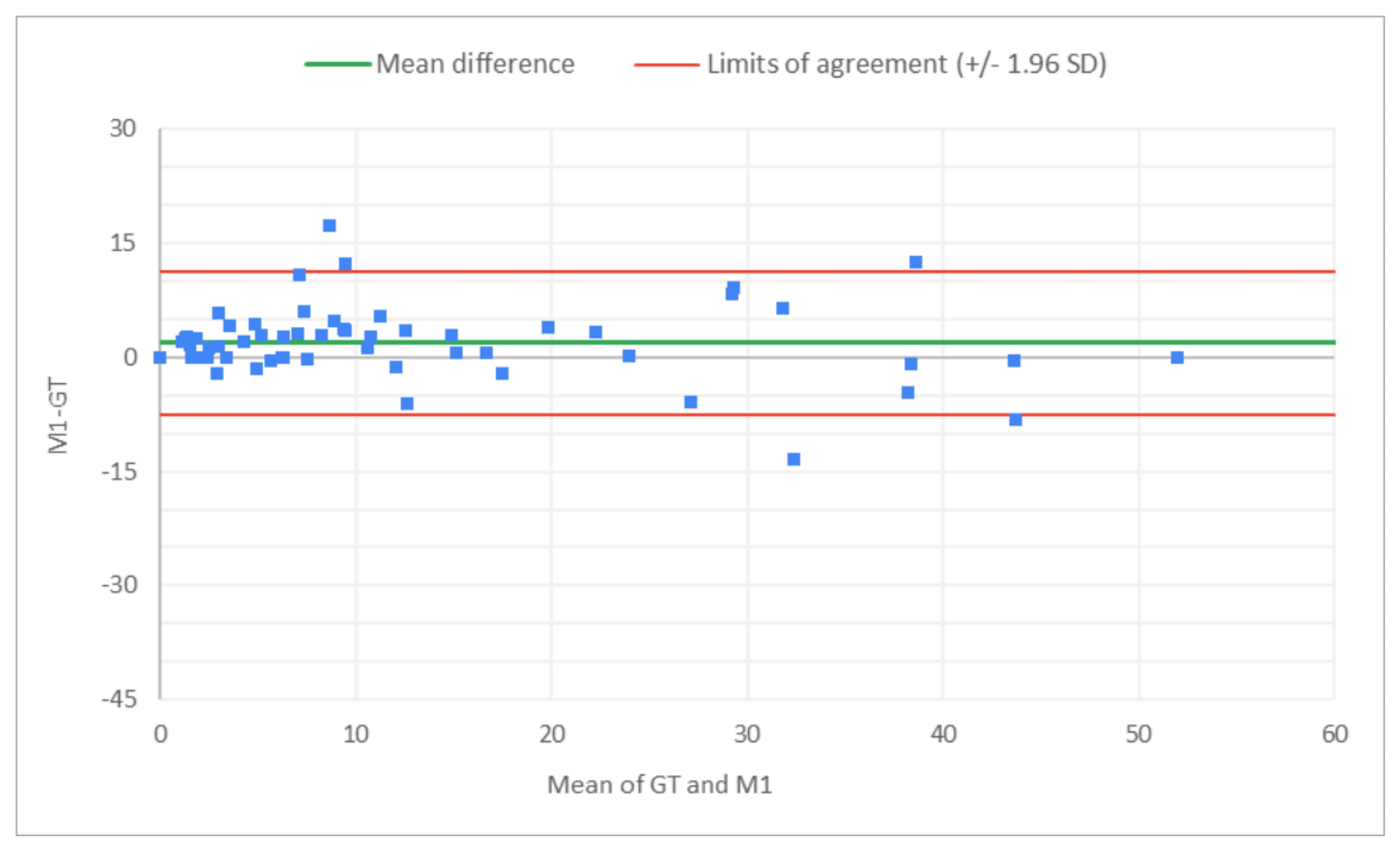
Bland-Altman plot for % SG: M1 (MDR of R1) vs. GT The x-axis represents the average of the two measurements, while the y-axis represents the difference between the two measurements. GT, ground truth; MDR, manual digital review; R1, Reviewer 1; SG, sclerotic glomeruli

**Figure 7:**
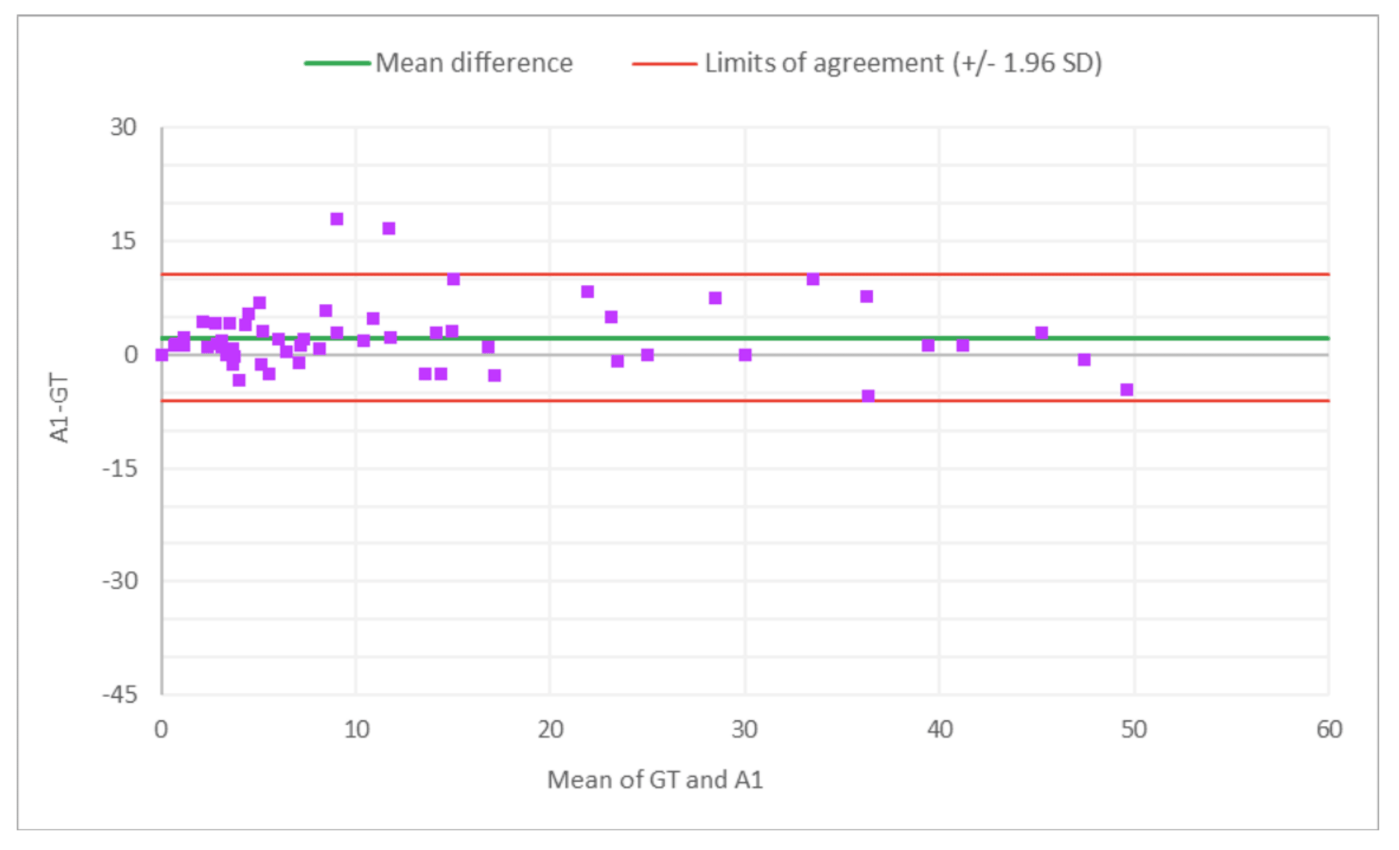
Bland-Altman plot for % SG: A1 (AAR of R2) vs. GT The x-axis represents the average of the two measurements, while the y-axis represents the difference between the two measurements. AAR, AI-assisted review; GT, ground truth; SG, sclerotic glomeruli; R2, Reviewer 2

**Figure 8:**
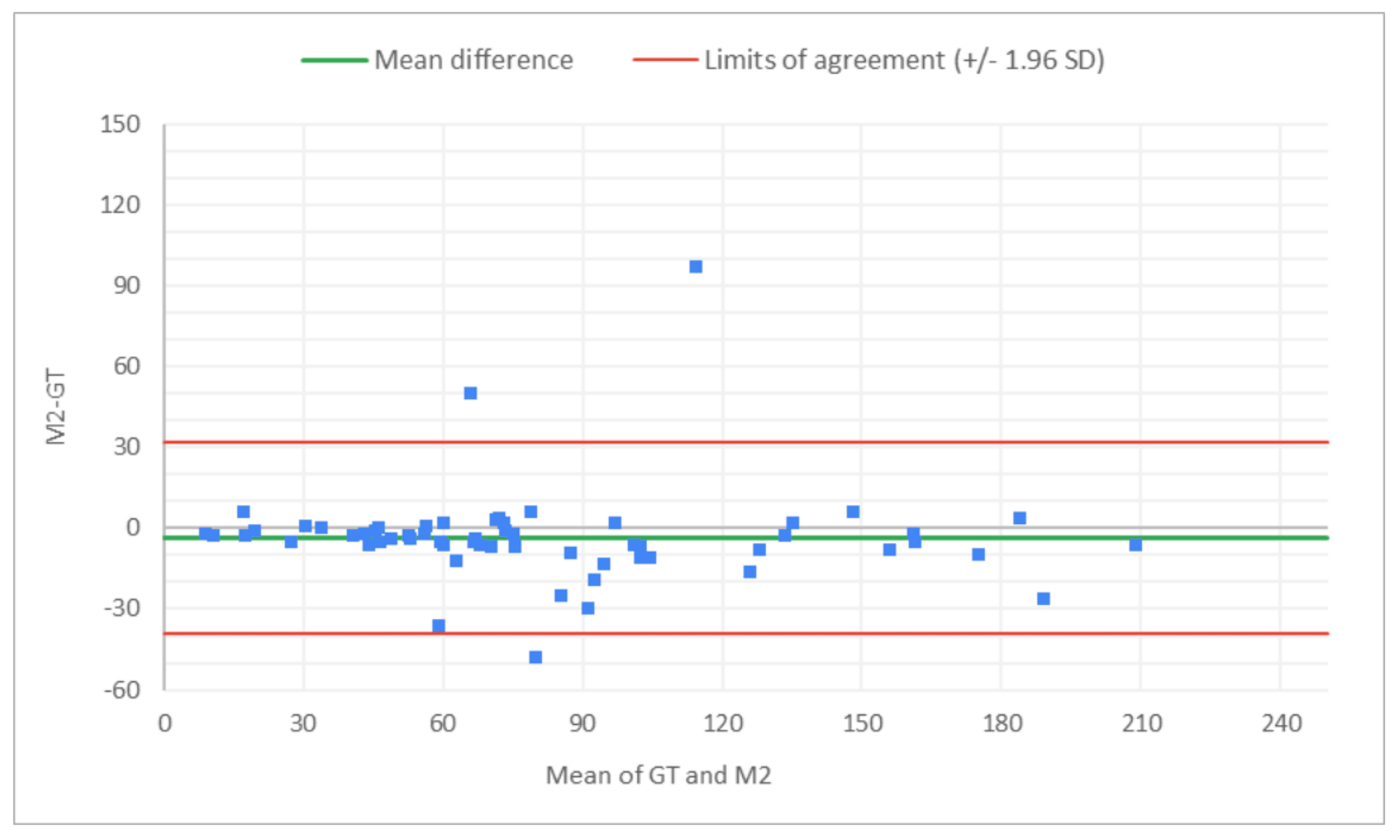
Bland-Altman plot for total glomeruli count: M2 (MDR of R2) vs. GT The x-axis represents the average of the two measurements, while the y-axis represents the difference between the two measurements. GT, ground truth; MDR, manual digital review; R2, Reviewer 2

**Figure 9:**
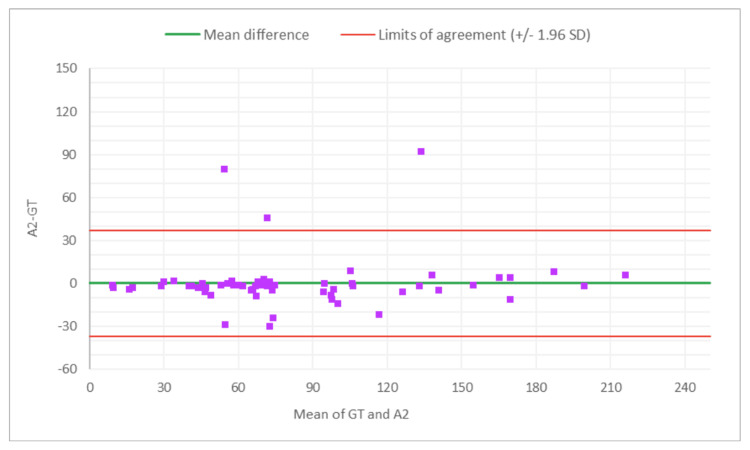
Bland-Altman plot for total glomeruli count: A2 (AAR of R2) vs. GT The x-axis represents the average of the two measurements, while the y-axis represents the difference between the two measurements. AAR, AI-assisted review; GT, ground truth; R2, Reviewer 2

**Figure 10:**
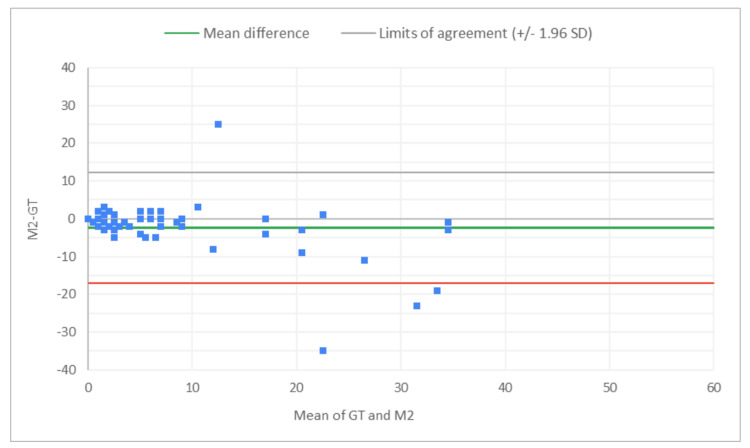
Bland-Altman plot for SG count: M2 (MDR of R2) vs. GT The x-axis represents the average of the two measurements, while the y-axis represents the difference between the two measurements. GT, ground truth; MDR, manual digital review; R2, Reviewer 2; SG, sclerotic glomeruli

**Figure 11:**
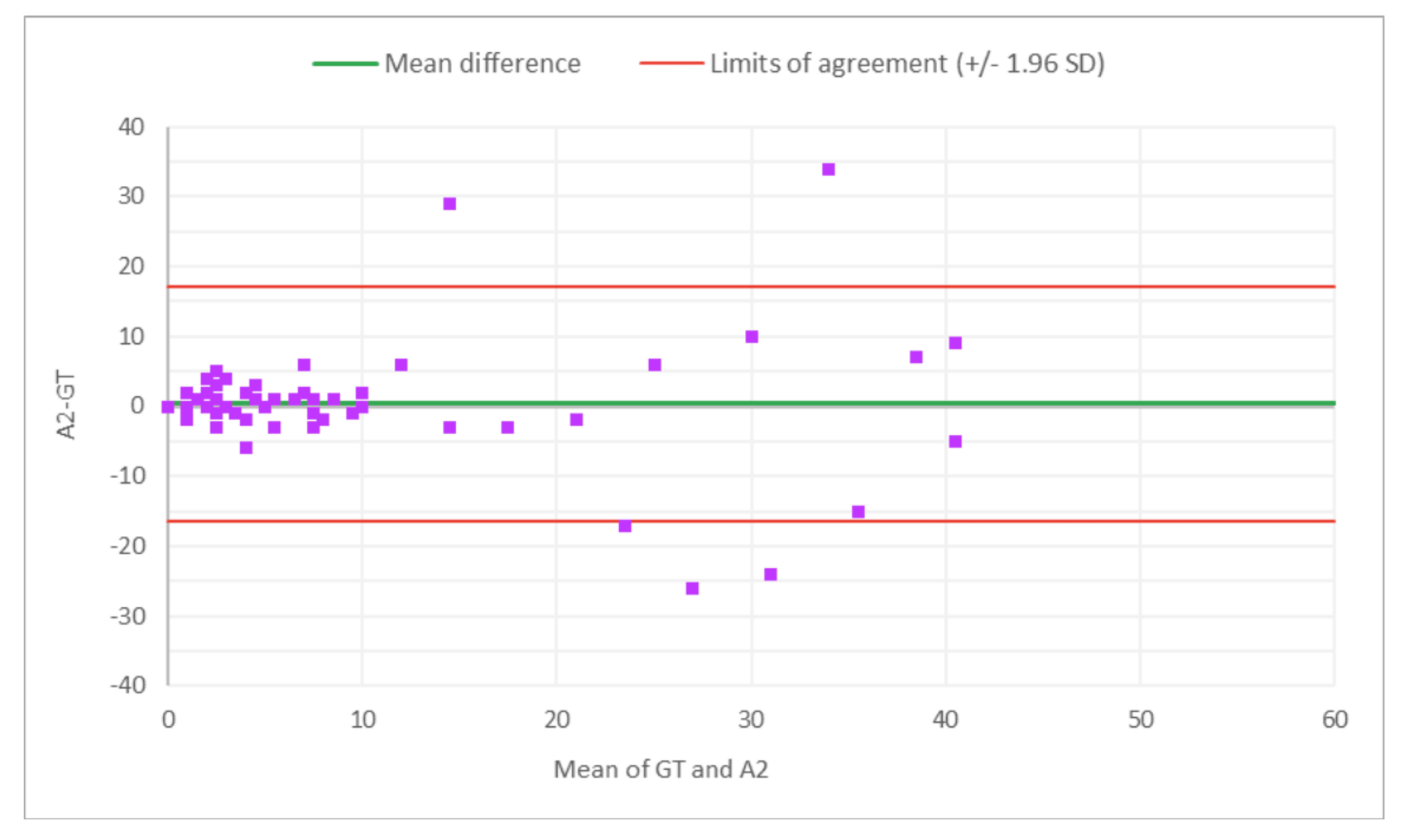
Bland-Altman plot for SG count: A2 (AAR of R2) vs. GT The x-axis represents the average of the two measurements, while the y-axis represents the difference between the two measurements. GT, ground truth; AAR, AI-assisted review; SG, sclerotic glomeruli; R2, Reviewer 2

**Figure 12:**
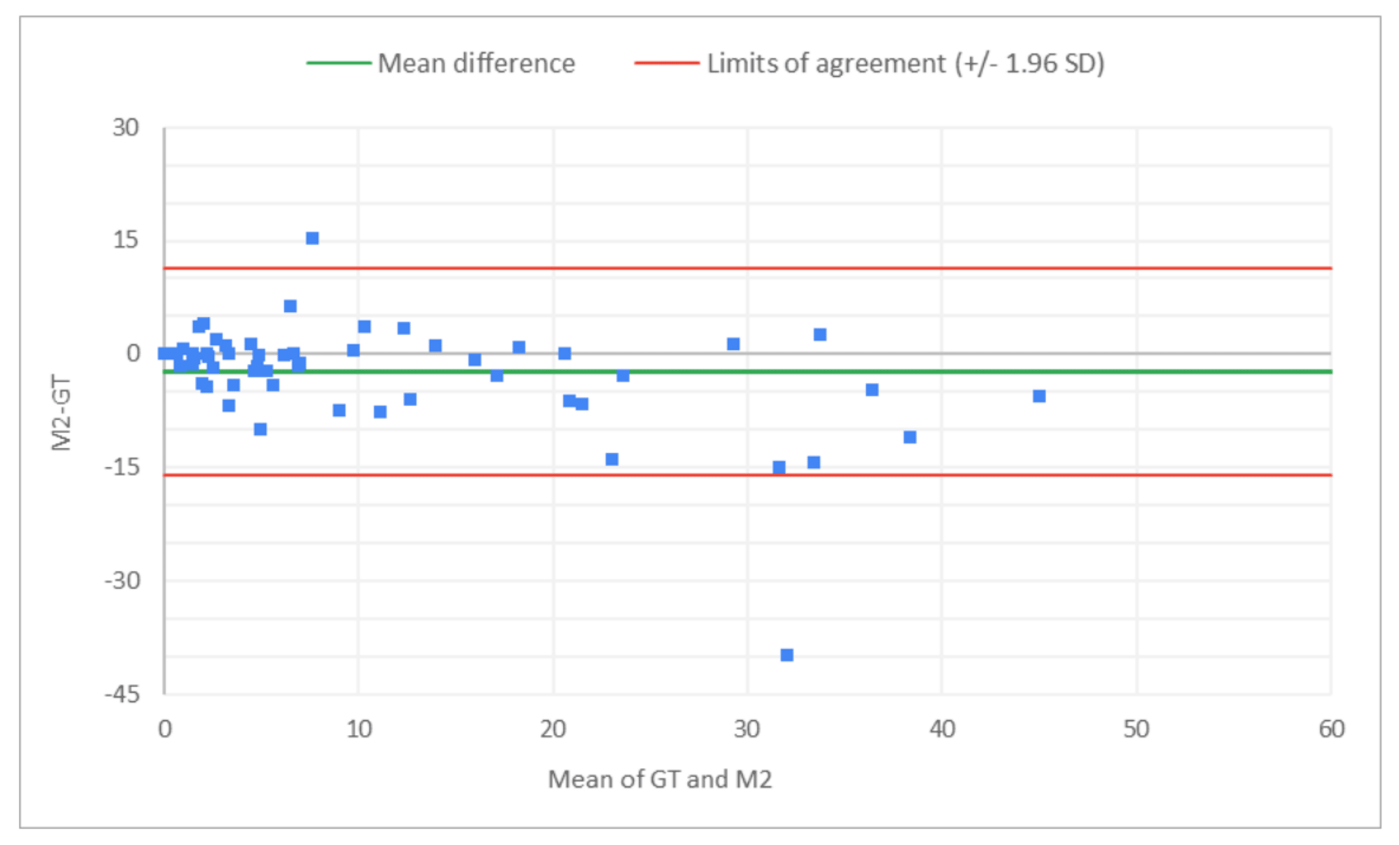
Bland-Altman plot for % SG: M2 (MDR of R2) vs. GT The x-axis represents the average of the two measurements, while the y-axis represents the difference between the two measurements. GT, ground truth; MDR, manual digital review; R2, Reviewer 2; SG, sclerotic glomeruli

**Figure 13:**
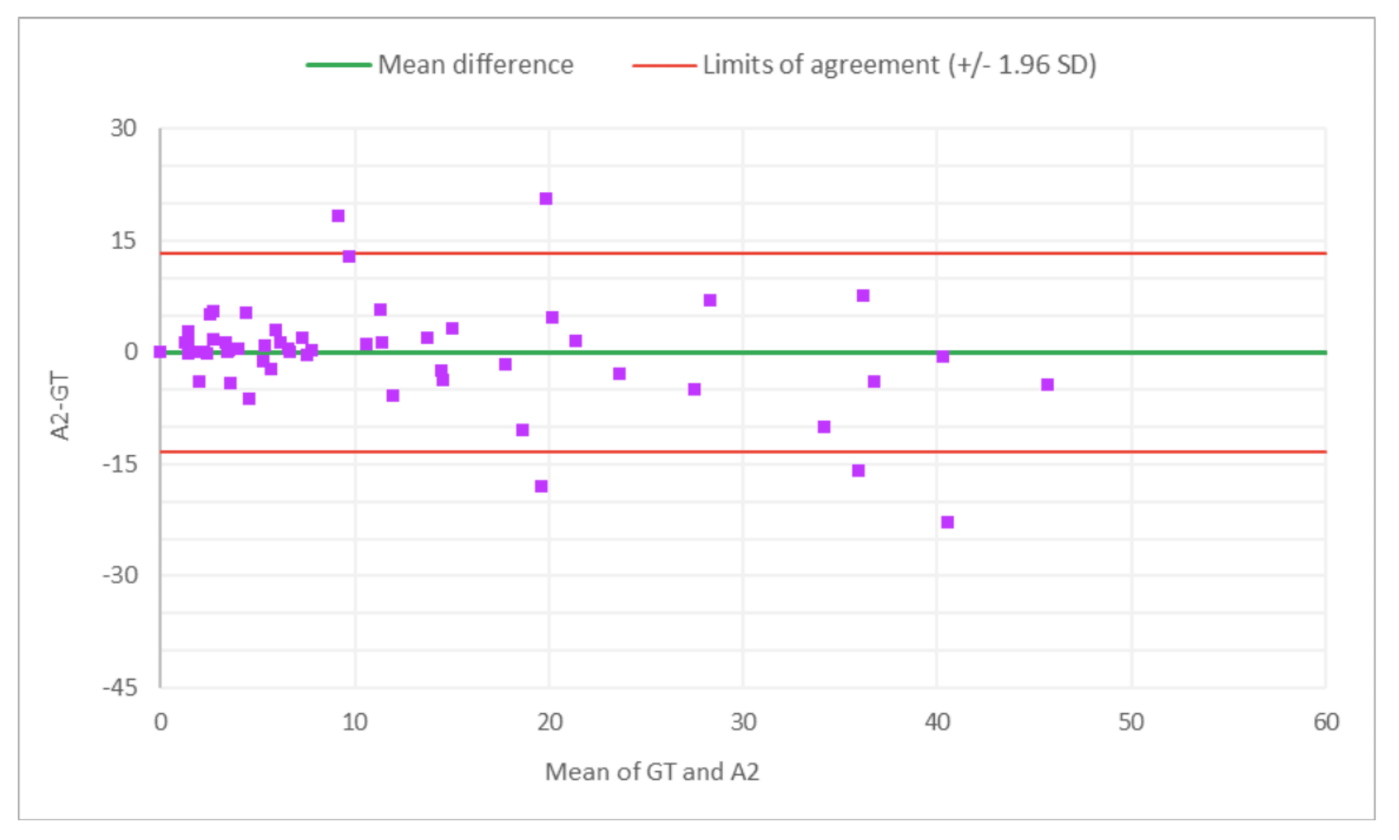
Bland-Altman plot for % SG: A2 (AAR of R2) vs. GT The x-axis represents the average of the two measurements, while the y-axis represents the difference between the two measurements. AAR, AI-assisted review; GT, ground truth; SG, sclerotic glomeruli; R2, Reviewer 2

The PCC and CCC values for AI alone for total glomeruli count and the rate of globally SG suggest moderate levels of linear correlation and agreement with the GT, indicating the potential of AI analysis alone in the kidney biopsy review process for organ transplant viability assessment. Across reviewers, PCC and CCC values for the rate of SG improved with AAR compared to the MDR, indicating stronger agreement with the expert review when AI assistance was used. A Bland-Altman analysis evaluated the level of agreement between MDR, AAR, and GT, by visualizing bias and limits of agreement (Figures 2-13).

Figures 2-7 show the agreement between the R1 and GT review results. The x-axis represents the average of the two measurements, while the y-axis represents the difference between the two measurements. The mean differences between M1 (MDR of R1) vs. A1 (AAR of R1) and GT for total glomeruli count, SG, and SG rate are 12.02 vs. 8.45, 3.0 vs. 3.0, and 1.92 vs. 2.23, respectively. This indicates that AAR aligns more closely with the GT assessments. The intervals between the upper and lower limits of agreement in the plots show the range within which 95% of the differences between R1 and GT fall. The limits of agreement across parameters for both M1 and A1 are relatively wide, suggesting some variability in the differences between R1 and GT across the range of measurements. No systematic bias was observed, as differences were scattered evenly across the mean difference line.

Figures 8-13 show the level of agreement between the R2 and GT review results. The mean differences between M2 (MDR of R2) vs. A2 (AAR of R2) and GT for total glomeruli count, SG, and SG rate are -3.53 vs. 0.2, -2.32 vs. 0.43, and -2.39 vs. 0.08, respectively. This indicates that the AAR better aligns with the GT assessments. The intervals between the upper and lower limits of agreement in the plots show the range within which 95% of the differences between R1 and GT fall. The limits of agreement across parameters for both M2 (MDR of R2) and A2 (AAR of R2) are relatively wide, which suggests that there is some variability in the differences between R2 and GT across the range of measurements. No systematic bias was observed, as differences were scattered evenly across the mean difference line.

## Discussion

This pilot study demonstrates the significant potential of AAR to enhance the efficiency and consistency of kidney biopsy evaluation for deceased donor organ transplantation [5,6]. Our results show that AAR significantly reduced review time while maintaining or improving agreement with GT compared to MDR. These findings suggest that AI-driven pathology can support transplant workflows by reducing evaluation time and improving standardization across viewers. 

The primary analysis revealed that AAR generally exhibited higher R² values than MDR when compared to GT for R1, indicating stronger correlation and improved agreement across key histopathological parameters. However, paired t-tests showed no statistically significant difference in the mean measurements between AAR and MDR for any parameters for R1. This suggests that while AAR’s measurements were more strongly correlated with the GT, the average values obtained by both methods were similar. For R2, MDR aligned slightly better with GT in terms of total glomeruli and SG count, as evidenced by slightly higher R² values. In contrast to R1, paired t-tests for R2 revealed statistically significant differences in the mean measurements between AAR and MDR for all parameters. Despite these differences in measurements for R2, the percentage of SG, an essential metric for kidney viability assessment, did not show a significant difference between the two methods, as the R² values were nearly identical. This finding is clinically significant as it suggests that AAR can help standardize the assessment of critical parameters such as the rate of SG, potentially leading to more uniform decision-making in kidney allocation. Standardization of this parameter across different institutions and reviewers could mitigate interobserver variability, a known challenge in biopsy-based transplantation evaluation. 

One of the most notable findings of this study was the substantial reduction in review time with AAR. Across both reviewers, AAR reduced mean review time by 54.83% per slide and 48.33% per glomerulus compared to MDR. This significant improvement in efficiency has critical implications for deceased donor organ allocation. In the context of kidney transplantation, where prolonged cold ischemia time is associated with worse graft outcomes, a more rapid biopsy evaluation process could help reduce delays and optimize donor-recipient matching [7]. Faster pathology workflows could also improve logistics in high-volume transplant centers, potentially increasing the number of successfully transplanted kidneys by enabling real-time decision-making.

The concordance analysis demonstrated that AAR improved agreement with GT for the percentage of SG, as reflected in higher PCC and CCC values compared to MDR. These findings suggest that AAR may serve as a valuable assistive tool, particularly in settings where experienced transplant pathologists are not readily available. The ability of AAR to align closely with expert review suggests that it could help bridge the expertise gap for general pathologists or transplant physicians who may not routinely evaluate donor kidney biopsies [8]. By improving diagnostic reliability and reducing subjectivity, AI-assisted workflows could support wider adoption of standardized biopsy evaluation protocols, ensuring more equitable organ allocation across different institutions.

This indicates stronger agreement with expert review when AI assistance was utilized, suggesting that AAR could help bridge the gap between experienced transplant pathologists and general pathologists or transplant physicians who may be less familiar with kidney biopsy evaluation [9]. This aspect of AAR is particularly valuable in settings where specialized transplant pathologists may not be readily available, potentially expanding the pool of qualified reviewers and facilitating more widespread adoption of standardized biopsy evaluation practices.

Importantly, our study showed that AI alone demonstrated moderate levels of linear correlation and agreement with GT for total glomeruli count and the rate of SG. This finding underscores the potential of AI analysis in the kidney biopsy review process, particularly as a supportive tool for human reviewers. While the current study focused on the partnership between AI and human reviewers, these results suggest that future iterations of the AI system could potentially perform certain aspects of the review process autonomously, further streamlining the evaluation workflow.

The Bland-Altman analysis revealed that AAR is better aligned with GT than MDR across multiple parameters. While the limits of agreement were relatively wide, indicating some variability, there was no clear systematic bias, suggesting that AAR could provide consistent results across different reviewers. This consistency is crucial in the context of organ transplantation, where decisions often need to be made quickly and with a high degree of confidence.

While this study focused on deceased donor kidney biopsies, the principles and methodology of AAR could be extended to other solid organs, such as liver and lung transplantation [10-12]. AI-assisted pathology has the potential to support both pre-transplant donor assessments and post-transplant monitoring, including the detection of rejection and other graft-related pathologies [13]. Expanding AI applications in transplant pathology could help create a more standardized, efficient, and reproducible approach to biopsy evaluation across multiple organ systems.

Despite these promising results, it is important to acknowledge the limitations of this pilot study. This study was conducted at a single center with a relatively small sample size, which may limit the generalizability of the findings. Future multi-center studies with larger datasets are necessary to validate these results. While AAR improved standardization, individual reviewer variability persisted, highlighting the need for further refinements in AI training to accommodate different pathologist interpretations. Finally, the impact of AI-assisted workflows on transplant outcomes, including long-term graft survival and rejection rates, was not assessed in this study. Future prospective studies with extended follow-up will be crucial to determine the clinical significance of AI-driven pathology.

As AI models continue to evolve, future advancements may enable more comprehensive biopsy analysis, including the evaluation of fibrosis, assessment of blood vessel damage (particularly arterial damage), and the ability to highlight various pathophysiological changes beyond glomerular damage. This expansion of capabilities could provide a more comprehensive assessment of organ quality, potentially leading to more nuanced allocation decisions and improved matching of donors to recipients. In addition, AI-driven pathology can be further combined with genomic, proteomic, and transcriptomic data to enhance predictive analytics for transplant success. The ultimate goal is to develop AI-powered tools that can support pathologists with real-time, multimodal insights, leading to more precise and personalized transplant decision-making.

Integrating AAR into clinical practice will require careful consideration of regulatory and ethical implications [14,15]. Ensuring the transparency and interpretability of AI-assisted decisions will be crucial for maintaining trust in the transplantation process. Furthermore, ongoing training and education for pathologists and transplant physicians will be necessary to ensure optimal use of this technology.

In conclusion, this pilot study demonstrates the potential of AAR to revolutionize kidney biopsy evaluation in deceased donor organ transplantation. By reducing review time, improving accuracy, and potentially standardizing assessments, AAR could significantly impact organ allocation processes, ultimately leading to improved outcomes for transplant recipients. As we continue to refine and expand this technology, the partnership between human expertise and AI promises to address critical challenges in organ transplantation, potentially saving more lives and improving the quality of life for transplant recipients worldwide.

## Conclusions

This pilot study highlights the significant potential of AAR to enhance the efficiency and consistency of kidney biopsy evaluation in deceased donor organ transplantation. AAR was found to significantly reduce review time while maintaining or improving agreement with GT, indicating its potential to support transplant workflows and optimize donor-recipient matching. Furthermore, the improved standardization of key histopathological parameters, such as the rate of SG, could mitigate interobserver variability and facilitate more uniform decision-making in kidney allocation. Notably, AAR demonstrated stronger correlation and agreement with expert review, suggesting its potential to bridge the expertise gap for general pathologists or transplant physicians who may not routinely evaluate donor kidney biopsies. The integration of AAR into clinical practice could lead to a more standardized, efficient, and reproducible approach to biopsy evaluation across multiple organ systems. To fully realize this potential, future studies are necessary to validate these results, refine AI training to accommodate different pathologist interpretations, and assess the impact of AI-assisted workflows on transplant outcomes. Additionally, the continued development and expansion of AI-powered tools could provide real-time, multimodal insights, leading to more precise and personalized transplant decision-making. However, careful consideration of regulatory and ethical implications, as well as ongoing training and education for pathologists and transplant physicians, will be crucial for the successful integration of AAR into clinical practice, ultimately leading to improved outcomes for transplant recipients.
